# Place of Death, Care Intensity, Symptom Prevalence, and Symptom Management for Older Adults with Stroke at End of Life

**DOI:** 10.1093/geroni/igaf122.3641

**Published:** 2025-12-31

**Authors:** Hanna Ramsburg, Janice Hinkle, Meredith MacKenzie Greenle

**Affiliations:** University of Pennsylvania School of Nursing, Philadelphia, Pennsylvania, United States; Villanova University, Villanova, Pennsylvania, United States; Villanova University, Villanova, Pennsylvania, United States

## Abstract

Each year in the United States, 795,000 people are diagnosed with a new or recurrent stroke, resulting in 165,393 deaths. However, little is known about the quality of end-of-life (EOL) care in older adults diagnosed with stroke. This secondary analysis examined whether place of death and the intensity of care received in the last month of life were correlated with the prevalence and management of EOL symptoms in older adults with stroke, using data from the National Health and Aging Trends Study, a representative sample of Medicare beneficiaries. Logistic regression was used to identify whether place of death and care intensity received in the last month of life were associated with symptom prevalence and symptom management in patients diagnosed with stroke, adjustive for demographic covariates. Of older adults diagnosed with stroke (n = 257), 62.6% reported pain, 50.6% reported dyspnea, and 57.6% reported emotional distress in the last month of life. Older adults who died in the acute care setting were half as likely to report pain (OR = 0.51, p = 0.05), but twice as likely to report dyspnea (OR = 2.07, p = 0.05) and adequate dyspnea management (OR = 1.97, p = 0.05). Place of death was not associated with emotional distress (OR = 1.62, p = 0.05), pain management (OR = 0.67, p = 0.05), or emotional distress management (OR = 0.97, p = 0.05). Age (OR = 0.56, p = 0.05) and race/ethnicity (OR = 4.80, p = 0.05) were associated with care intensity, but symptom prevalence and management were not associated. Results suggest that older adults with stroke have distressing symptoms at EOL, but future innovative research is needed in the evaluation and management of symptoms at EOL.

